# Preclinical Evaluation of Discorhabdins in Antiangiogenic and Antitumor Models

**DOI:** 10.3390/md16070241

**Published:** 2018-07-19

**Authors:** Emily M. Harris, Jonathan D. Strope, Shaunna L. Beedie, Phoebe A. Huang, Andrew K. L. Goey, Kristina M. Cook, Christopher J. Schofield, Cindy H. Chau, Melissa M. Cadelis, Brent R. Copp, Kirk R. Gustafson, William D. Figg

**Affiliations:** 1 Molecular Pharmacology Section, Genitourinary Malignancies Branch, Center for Cancer Research, National Cancer Institute, National Institutes of Health, Bethesda, MD 20892, USA; emily.harris5050@gmail.com (E.M.H.); jonathan.strope@nih.gov (J.D.S.); shaunna.beedie@oncology.ox.ac.uk (S.L.B.); phoebe.huang@nih.gov (P.A.H.); andrewgoey@hotmail.com (A.K.L.G.); kristina.cook@sydney.edu.au (K.M.C.); chauc@mail.nih.gov (C.H.C.); 2 Department of Chemistry, Chemistry Research Laboratory, University of Oxford, Oxford OX1 5JJ, UK; christopher.schofield@chem.ox.ac.uk; 3 School of Chemical Sciences, University of Auckland, Auckland 1142, New Zealand; m.cadelis@auckland.ac.nz (M.M.C.); b.copp@auckland.ac.nz (B.R.C.); 4 Molecular Targets Program, Center for Cancer Research, National Cancer Institute, Frederick, MD 21702, USA; gustafki@mail.nih.gov

**Keywords:** discorhabdins, hypoxia, HIF, angiogenesis, marine natural products, alkaloids

## Abstract

Elements of the hypoxia inducible factor (HIF) transcriptional system, a key regulator of the cellular hypoxic response, are up-regulated in a range of cancer cells. HIF is fundamentally involved in tumor angiogenesis, invasion, and energy metabolism. Inhibition of the transcriptional activity of HIF may be of therapeutic benefit to cancer patients. We recently described the identification of two marine pyrroloiminoquinone alkaloids with potent activity in inhibiting the interaction between the oncogenic transcription factor HIF-1α and the coactivator protein p300. Herein, we present further characterization data for these two screening hits: discorhabdin H (**1**) and discorhabdin L (**2**), with a specific focus on their anti-angiogenic and anti-tumor effects. We demonstrated that only discorhabdin L (**2**) possesses excellent anti-angiogenic activity in inhibiting endothelial cell proliferation and tube formation, as well as decreasing microvessel outgrowth in the ex vivo rat aortic ring assay. We further showed that discorhabdin L (**2**) significantly inhibits in vivo prostate tumor growth in a LNCaP xenograft model. In conclusion, our findings suggest that discorhabdin L (**2**) represents a promising HIF-1α inhibitor worthy of further drug development.

## 1. Introduction

Hypoxia inducible factor (HIF) is a heterodimeric transcription factor and master regulator of oxygen homeostasis in animals. Vascular endothelial growth factor (regulating angiogenesis), erythropoietin (regulating the production of red blood cells), and glycolytic enzymes are some of the vast numbers of HIF-1 target genes that are activated in response to low oxygen levels [1]. HIF-1 is comprised of an inducibly-expressed HIF-1α subunit and a constitutively-expressed HIF-1β subunit. Under hypoxic conditions, HIF-1α is stabilized, translocates into the nucleus, dimerizes with HIF-1β, and binds to cognate hypoxia response elements (HREs). The HIF αβ-heterodimer then recruits the p300/CREB-binding protein (CBP) family of coactivators to promote transcription of target genes in a context-dependent manner [2,3,4,5].

**Figure 1:**
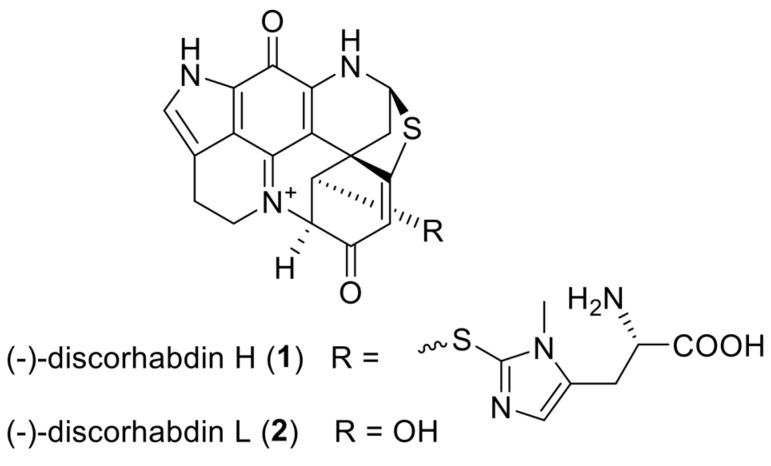
Chemical structures of discorhabdins H (**1**) and L (**2**).

Inhibition of HIF-1 represents an attractive therapeutic strategy for targeting hypoxia, a hallmark of many solid tumors, and tumor angiogenesis. One promising approach for directly inhibiting HIF-1 activity is by disrupting the tight binding between HIF-1α and p300 [6,7,8,9]. Previously, our laboratory developed an in vitro fluorescence binding assay that can be used in a high-throughput screen to identify small-molecule inhibitors of HIF-1α, through inhibiting the binding interaction between the C-terminal transactivation domain (CTAD) of HIF-1α and the cysteine/histidine-rich 1 (CH1) domain of p300 [7]. Using our HIF-1α/p300 assay, we performed high-throughput screening of extracts and prefractioned samples from the National Cancer Institute’s Natural Products Repository. This effort led to the discovery of a series of pyrroloiminoquinone alkaloids including discorhabdin and makaluvamine alkaloids, originating from a *Latrunculia* sp. of marine sponge, as potential HIF-1α/p300 inhibitors [9,10]. Discorhabdins contain ring structures unique in natural products, that is, azacarbocyclic spirocyclohexanone and pyrroloiminoquinone redox active core structures, and they exhibit a plethora of biological properties, including strong cytotoxic, antimicrobial, antiviral, antimalarial, and immunomodulatory effects [11]. Due to their wide range of biological activities, research on the isolation, structural determination, and synthesis of these alkaloids has attracted considerable attention [12]. We recently identified a novel molecular mechanism of discorhabdins which involves targeting the HIF-1α/p300 complex. We described a cohort of marine pyrroloiminoquinone alkaloids and evaluated their biological effects in various cancer cell lines, including their cytotoxicity and inhibitory activity against HIF-1α transcription and expression of its downstream target, vascular endothelial growth factor (VEGF) [9]. Here, we present the preclinical characterization of two lead compounds: discorhabdin H (**1**) and discorhabdin L (**2**) (Figure 1), with a specific focus on their anti-angiogenic and anti-tumor effects.

## 2. Results and Discussion

**Figure 2:**
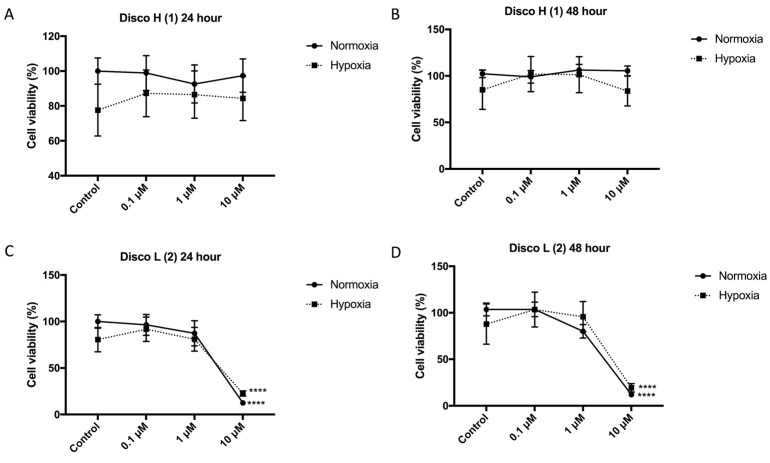
Effect of discorhabdins on endothelial cell proliferation. Human umbilical vein endothelial cells (HUVECs) were treated with discorhabdin H (**1**) or discorhabdin L (**2**) at various concentrations under normoxic or hypoxic (1% O_2_) conditions for 24 h (**A**,**C**) and 48 h (**B**,**D**). Cell proliferation was assessed using a CCK-8 assay. The result is representative of three independent experiments performed in triplicate, with cell proliferation expressed as a percentage of untreated normoxia controls ± SEM (**** *p* < 0.0001).

The aim of our study was to further evaluate and functionally characterize the two most potent discorhabdin compounds, discorhabdin H (**1**) and discorhabdin L (**2**), identified in our previous screen [9]. Given that the compounds demonstrated inhibition of HIF-1α activity and a decrease in secretion of the HIF target protein VEGF (both essential for tumor angiogenesis), we first determined the inhibitory effect of the discorhabdins on endothelial cell function and blood vessel formation. The cytotoxicity of the compounds was assessed on human umbilical vein endothelial cells (HUVECs). HUVECs were incubated with increasing concentrations of the discorhabdins (0.1, 1, and 10 μM) in the presence of normoxia and hypoxia (1% O_2_). Discorhabdin H (**1**) exhibited minimal toxicity to endothelial cells at all test concentrations (IC_50_ > 10 μM), regardless of hypoxic/normoxic conditions or treatment duration (Figure 2A,B). In contrast, treatment with 10 μM discorhabdin L (**2**) significantly reduced HUVEC cell proliferation in both normoxic and hypoxic conditions at both 24 and 48 h (IC_50_ ~ 5 μM, *p* < 0.0001) (Figure 2C,D). The sensitivity of endothelial cells to discorhabdin L (**2**) is similar to that of a previous study reporting pyrroloiminoquinone alkaloids to be highly cytotoxic for HCT 116 cells, with IC_50_ values in the lower micromolar range [13]. Thus, discorhabdins demonstrate differential cytotoxicity that is cell-type dependent.

**Figure 3:**
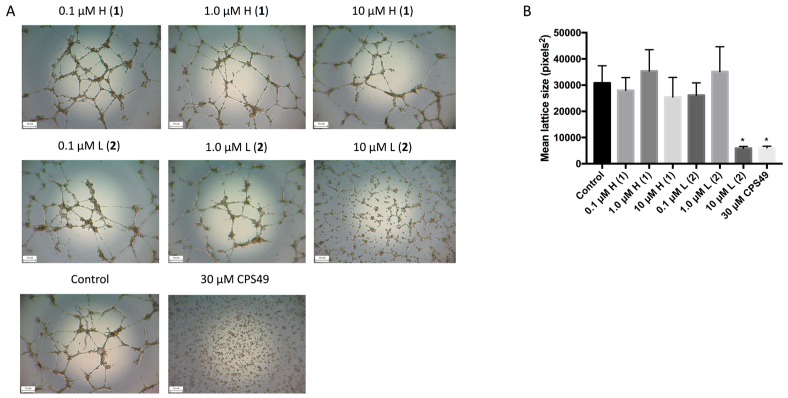
Effects of discorhabdins on endothelial tube formation. An in vitro angiogenesis assay was used with the ECMatrix system and HUVECs were plated in 96-well plates precoated with ECMatrix (50 μL/well). Cells were treated with 0.1 μM, 1.0 μM, and 10 μM of discorhabdin H (**1**) or discorhabdin L (**2**), 30 μM CPS49 (positive control) or media control for 18 h. Results represent three independent experiments performed in triplicate. (**A**) Representative images of tubule formation for each treatment group are shown (images were taken at 4× magnification); (**B**) Quantitative data of tube formation using ImageJ. Data are expressed as the mean ± SEM of the HUVEC mesh size (* *p* < 0.05).

We then investigated the anti-angiogenic activity of the discorhabdins (**1** and **2**) on endothelial cell tube formation. HUVECs can form hollow tube-like structures when cultured upon biological gels, such as ECMatrix (EMD Millipore, Darmstadt, Germany). The formation of the tubules can then be used as a simple in vitro measurement of angiogenesis, with the extent of inhibition corresponding to the anti-angiogenic effects of the compounds. Following treatment with either the positive control CPS49, a well characterized potent anti-angiogenic compound [14], or 10 μM discorhabdin L (**2**), tubule formation was significantly inhibited (Figure 3). This effect was not observed following treatment with any concentration of discorhabdin H (**1**).

**Figure 4:**
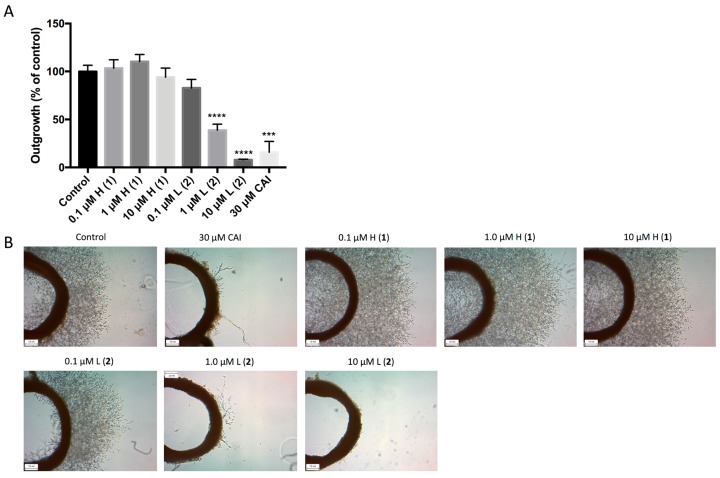
Effects of discorhabdins on rat aortic ring microvessel outgrowth. Rat aortic rings were dissected and plated within Matrigel in 24-well plates. Rings were treated with media containing VEGF to stimulate microvessel outgrowth. The following day, the media was removed and rings were treated with 0.1 μM, 1.0 μM, and 10 μM of discorhabdin H (**1**) or discorhabdin L (**2**), 30 μM CAI (positive control) or media depleted of VEGF for 5 days. Results are representative of four independent experiments performed in triplicate. (**A**) Outgrowth is expressed as percent outgrowth relative to that of untreated control ± SEM (*** *p* < 0.001, **** *p* < 0.0001); (**B**) Representative images of outgrowths for each treatment group are shown (taken at 4× magnification).

We further examined the effect of the two lead compounds on microvessel formation using the three-dimensional ex vivo rat aortic ring model [15], which recapitulates the complexities of angiogenesis and combines the advantages of in vitro and in vivo models. Outgrowth of microvessels in this system was significantly reduced following treatment with both 1 μM and 10 μM discorhabdin L (**2**), which resulted in >50% and >90% inhibition of angiogenesis, respectively (Figure 4). Treatment with discorhabdin H (**1**) did not result in a decrease in microvessel outgrowth.

**Figure 5:**
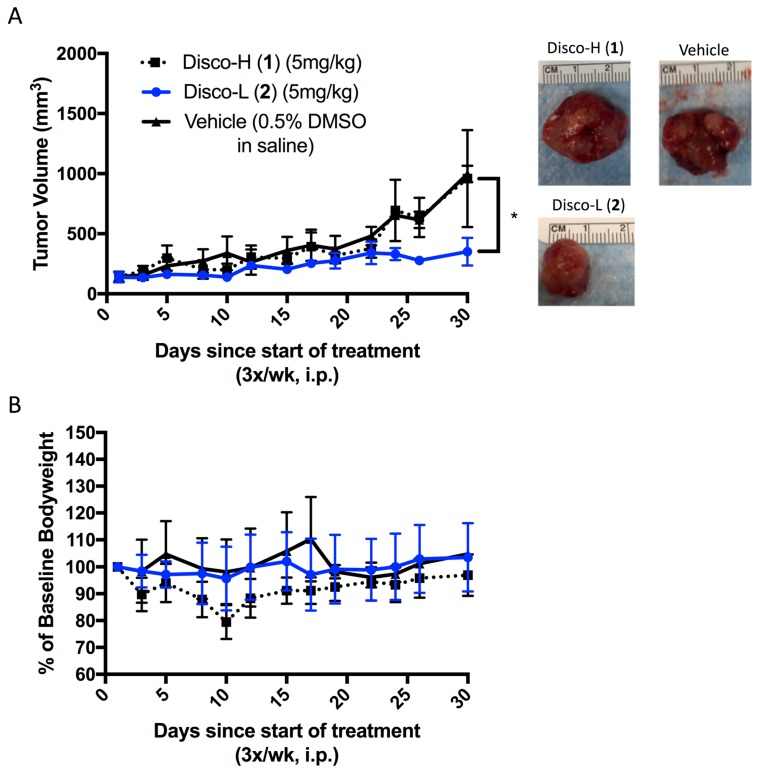
Effects of discorhabdins in vitro. Six-week old, male, severe combined immunodeficiency (SCID) mice were injected with 100 μL of LNCaP/Matrigel bolus (5,000,000 cells/mouse). Once tumors were palpable, mice were treated 3×/week with either drug vehicle (0.5% DMSO in saline), discorhabdin H (**1**), or discorhabdin L (**2**) at 5 mg/kg for 4 weeks (*n* = 3 per treatment group). (**A**) Tumor volumes throughout treatment are shown, measured 3×/week (* *p* < 0.05). Representative images of excised tumors at the end of treatment are shown; (**B**) Mice were weighed daily. Percent of baseline bodyweight between the three treatment groups is shown.

Finally, we evaluated the in vivo efficacy of the discorhabdins (**1** and **2**) in a prostate cancer xenograft model. We selected the LNCaP cell line based on prior in vitro data with the discorhabdins [9]. LNCaP cells were implanted subcutaneously into severe combined immunodeficiency mice. Once tumors became palpable, vehicle or the discorhabdin compounds were administered for 4 weeks by 3×/weekly intraperitoneal injections. Only discorhabdin L (**2**) significantly inhibited LNCaP tumor growth compared to the vehicle control (Figure 5A). No significant reduction in body weight was observed in LNCaP-bearing mice treated with the lead compounds (Figure 5B).

In summary, we have evaluated the anti-angiogenic and anti-tumor effects of two lead compounds, discorhabdins H (**1**) and L (**2**), previously identified by us as inhibitors of HIF-1α/p300 binding. We demonstrated that discorhabdin L (**2**) possesses excellent anti-angiogenic activity in inhibiting endothelial cell proliferation and tube formation as well as decreasing microvessel outgrowth in the ex vivo rat aortic ring assay. We further demonstrated that discorhabdin L (**2**) significantly inhibits in vivo prostate tumor growth in a LNCaP xenograft model (Figure 5). Presently, we are not certain of the precise in vivo mechanism of action of discorhabdin L (**2**). Given their complex and redox active heterocyclic ring structure, it is possible that discorhabdins have other molecular mechanisms of action in addition to disrupting the CBP/p300:HIF-1 interaction. Nonetheless, our results, coupled with those previously reported, show that discorhabdins inhibit HIF-1α transcriptional activity and decrease VEGF protein secretion [9]. Thus, we conclude that discorhabdin L (**2**) is a promising therapeutic lead for inhibiting HIF-1 transcriptional activity that warrants further evaluation as a potential anti-tumor and anti-angiogenic agent. Studies are currently underway to determine other HIF-1α downstream targets and effector molecules of discorhabdin L (**2**), and to determine whether it possesses other pleiotropic effects attributed to this class of compounds, such as immunomodulatory activity. Compound availability is one of the limitations of our study since the discorhabdins were isolated from a marine sponge; therefore, there is clearly a need to develop synthetic routes to produce a stable supply of discorhabdins and novel analogs for further evaluation and preclinical characterization. Future studies of the in vivo efficacy of **2** in other cancer models are also warranted, including optimization of dose and schedule. Dose and schedule are likely to be important given the pleiotropic and context-dependent nature of the HIF-mediated hypoxic response, including with respect to the role of the interaction of HIF with CBP/p300 [5]. In conclusion, our findings demonstrate that discorhabdin L (**2**) represents a promising HIF-1α inhibitor worthy of further drug development.

## 3. Materials and Methods

### 3.1. Cell Culture

HUVEC cells (Lonza, Walkersville, MD, USA) were cultured in endothelial growth medium (EGM)-plus media (Lonza, Walkersville, MD, USA) and used before passage 12. To split, the cells were detached using TryplE Express (ThermoFisher Scientific, Waltham, MA, USA), spun at 300 rpm, and resuspended in EGM-plus media. LNCaP cells were purchased from ATCC (Manassas, VA, USA) and maintained in phenol red-free RPMI 1640 medium purchased from Gibco (Gaithersburg, MD, USA) supplemented with 10% FBS and 1% Pen Strep (100 U/mL penicillin + 100 μg/mL streptomycin). Cells were cultured in 5% CO_2_ and 95% air at 37 °C. For hypoxia experiments, cells were placed in a hypoxia chamber (BioSpherix, Parish, NY, USA) set at 1% O_2_.

### 3.2. Compounds

The discorhabdin compounds used in this study were previously described [9]. Each compound was dissolved in DMSO, aliquoted, then frozen at −20 °C until required for experimental use.

### 3.3. Cytotoxicity Assay on HUVECs

HUVECs were seeded in 96-well plates (Corning, Corning, NY, USA) at a concentration of 600,000 cells/well. Twenty-four hours later, cells were treated with fresh medium containing either discorhabdin H (**1**), discorhabdin L (**2**) at varying concentrations (0.1 μM, 1.0 μM, or 10 μM) or vehicle (dimethyl sulfoxide, DMSO) control. Each concentration was tested in triplicate, and experiments were performed in triplicate. Cells were incubated for 24 or 48 h in normoxic or hypoxic conditions, and then 10 μL of cell counting kit (CCK)-8 solution (Dojindo, Rockville, MD, USA) was added to each well. After incubation for 2 h at 37 °C, absorbance was read at 450 nm on a SpectraMax M2 plate reader (Molecular Devices, San Jose, CA, USA). The fluorescent signal of each sample was normalized to the average signal of the DMSO-treated controls to calculate percent cell viability.

### 3.4. Endothelial Cell Tube Formation Assay

The in vitro angiogenesis assay kit was purchased from EMD Millipore (Darmstadt, Germany). Briefly, ECMatrix (50 μL/well) was plated to a 96-well plate and left to set for 30 min. HUVECs were plated atop the gel (50,000 cells/well) and treated with 0.1 μM, 1.0 μM, and 10 μM of discorhabdin H (**1**) and discorhabdin L (**2**), and 30 μM CPS49 (positive control). Wells were imaged after 18 h of incubation. Tubule formation was quantified using ImageJ.

### 3.5. Rat Aortic Ring Assay of Angiogenesis

Rats were sacrificed and the descending aortas were dissected into 1 mm sections. Matrigel (250 μL) (Growth Factor Reduced, Corning, Corning, NY, USA) was added to the wells of 24-well plates and incubated for 1 h to set. Rings were added to the wells along with another 250 μL of Matrigel. Rings were incubated overnight with EGM-II media containing VEGF (Lonza, Walkersville, MD, USA). The next day, media was replaced with EGM-II without VEGF and containing discorhabdin H (**1**) or discorhabdin L (**2**) (0.1 μM, 1.0 μM, or 10 μM), 30 μM carboxyamidotriazole (CAI, a known angiogenesis inhibitor as the positive control), or no treatment. Rings were incubated for 5 days and then imaged. Due to the large sizes of the aortic rings, 4 separate images were taken for each ring, each of a quarter of the ring. These 4 images were stitched together using Nikon NIS-Elements software (Version 4.2, Nikon Instruments, Melville, NY, USA) in order to obtain a full image of each ring. Microvessel outgrowth was then quantified using Adobe Photoshop (Version 18.1.0, San Jose, CA, USA). Additionally, partial representative images of the rings were taken at 4× magnification.

### 3.6. In Vivo Efficacy Study

Six-week old, male, severe combined immunodeficiency (SCID) mice were obtained from the NCI-Frederick Animal Production Area. LNCaP cells were cultured in maintenance media and harvested when they reached 80% confluency. Cells were washed with sterile phosphate buffered saline (Gibco), counted, and resuspended in Matrigel (5,000,000 cells/mouse) (Corning, Corning, NY, USA). LNCaP cells in the Matrigel bolus were injected subcutaneously in the rear flank of each SCID mouse (100 μL/mouse). Mice were monitored and tumors were measured with a caliper. When tumors became palpable, animals were randomized into 3 groups: each group was treated 3 times a week with intraperitoneal bolus injections of either the drug vehicle (0.5% DMSO in saline) or discorhabdin H (**1**) or discorhabdin L (**2**) at 5 mg/kg. This dose was the maximum tolerated dose determined in a previous study that was shown to be safe wherein mice did not show any outward toxicities with compound administration (data not shown). Mice were treated for 4 weeks. Tumor measurements used to calculate tumor volume (tumor volume = tumor length × tumor width × tumor height × π/6) were taken three days per week and mice were weighed daily. Tumors were excised on the last day of study and harvested tissue samples were snap frozen in liquid nitrogen.

The National Cancer Institute (NCI) is accredited by the Association for Assessment and Accreditation of Laboratory Animal Care (AAALAC) International and follows the Public Health Service (PHS) Policy for the Care and Use of Laboratory Animals. Animal care was provided in accordance with the Guide for the Care and Use of Laboratory Animals. The study protocol was approved by the NCI Animal Care and Use Committee.

### 3.7. Statistical Analysis

Analysis was conducted using GraphPad Prism software (Version 7, GraphPad Software, La Jolla, CA, USA). Statistical significance was assessed using two-tailed Students t-tests or ANOVA analyses and error bars represent standard error of the mean (SEM).
